# Predicting IDH Mutation Status in Low-Grade Gliomas Based on Optimal Radiomic Features Combined with Multi-Sequence Magnetic Resonance Imaging

**DOI:** 10.3390/diagnostics12122995

**Published:** 2022-11-30

**Authors:** Ailing He, Peng Wang, Aihua Zhu, Yankui Liu, Jianhuan Chen, Li Liu

**Affiliations:** 1 Big Data Center, Affiliated Hospital of Jiangnan University, Wuxi 214122, China; 2 Department of Radiology, Affiliated Hospital of Jiangnan University, Wuxi 214122, China; 3 Department of Neurosurgery, Affiliated Hospital of Jiangnan University, Wuxi 214122, China; 4 Department of Pathology, Affiliated Hospital of Jiangnan University, Wuxi 214122, China; 5 Laboratory of Genomic and Precision Medicine, Wuxi School of Medicine, Jiangnan University, Wuxi 214122, China

**Keywords:** glioma, multi-sequence MRI, radiomics, IDH, machine learning

## Abstract

The IDH somatic mutation status is an important basis for the diagnosis and classification of gliomas. We proposed a “6-Step” general radiomics model to noninvasively predict the IDH mutation status by simultaneously tuning combined multi-sequence MRI and optimizing the full radiomics processing pipeline. Radiomic features (*n* = 3776) were extracted from multi-sequence MRI (T1, T2, FLAIR, and T1Gd) in low-grade gliomas (LGGs), and a total of 45,360 radiomics pipeline were investigated according to different settings. The predictive ability of the general radiomics model was evaluated with regards to accuracy, stability, and efficiency. Based on numerous experiments, we finally reached an optimal pipeline for classifying IDH mutation status, namely the T2+FLAIR combined multi-sequence with the wavelet image filter, mean data normalization, PCC dimension reduction, RFE feature selection, and SVM classifier. The mean and standard deviation of AUC, accuracy, sensitivity, and specificity were 0.873 ± 0.05, 0.876 ± 0.09, 0.875 ± 0.11, and 0.877 ± 0.15, respectively. Furthermore, 14 radiomic features that best distinguished the IDH mutation status of the T2+FLAIR multi-sequence were analyzed, and the gray level co-occurrence matrix (GLCM) features were shown to be of high importance. Apart from the promising prediction of the molecular subtypes, this study also provided a general tool for radiomics investigation.

## 1. Introduction

Isocitrate dehydrogenase (IDH) is an important molecular diagnosis bias in low-grade gliomas (LGGs) [1,2]. For LGGs, the IDH phenotype affects not only the degree of tumorigenesis, but also the patients’ clinical outcome. Usually, patients with IDH mutations have a better prognosis than IDH wild-type patients [3,4,5]. The 2016 World Health Organization Classification of Tumors of the Central Nervous System (2016 CNS WHO) clarified the importance of IDH mutation status in the diagnosis of gliomas [6]. Furthermore, the new version, the 2021 CNS WHO [7], emphasized the value of molecular diagnosis and pathological features in tumor classification, which could provide an evaluation basis for early diagnosis and individualized treatment of gliomas.

Machine learning-based radiomics is an extensive research topic in predicting tumor subtypes [8,9,10,11,12], which is widely applied to disease assessment because quantitative features can be extracted from conventional imaging (CT, MRI, PET, etc.). Emerging studies have demonstrated promising values for radiomics in predicting IDH mutation status [13,14,15,16,17,18,19,20,21], which enables a noninvasive, real-time, and reproducible acquisition mechanism for glioma subtype research. In 2020, the linear classification model was used by Kim et al. [14] to predict IDH mutation status with multi-sequence MRIs including diffusion weighted imaging (DWI), contrast-enhanced T1-weighted imaging (CE-T1WI), fluid-attenuated inversion recovery (FLAIR), and apparent diffusion coefficient (ADC). It proved that multiparametric MRIs performed better in IDH mutation status prediction, and ADC features were significant. Santinha J et al. [18] combined T1-weighted (T1), T2-weighted (T2), and FLAIR multi-sequence MRIs with the traditional radiomics model to predict IDH mutation status with different features acquisition environment. They verified that the feature screening algorithm has better performance and robustness in the prediction model. Li et al. [19] reported the different settings in their radiomics pipeline based on T1, T2, FLAIR, and post-contrast T1-weighted (T1Gd) multi-sequence MRIs. Their findings provided a better understanding of radiomics model development and interpretability. Choi, Y.S. et al. [21] used an automatic segmentation method for multi-parametric MRI images (T1, T2, FLAIR) to predict IDH mutation status, and the TCGA database was used for external validation. They found that the hybrid model of automatic segmentation performed well in IDH prediction. However, the diagnostic performances achieved through tuning different combinations of multi-sequence MRIs and investigating different settings in the radiomics pipeline simultaneously remain unexplored.

To solve these problems, we focus on two aspects in this paper. First, based on the traditional machine learning-based radiomics technology [8,9,10,11,12], we proposed a “6-Step” general radiomics model, which can investigate both the optimal combined multi-sequence MRI and the most appropriate settings in the radiomics pipeline simultaneously, to noninvasively distinguish the IDH mutation status. The “6-Step” general radiomics model can be applied to different images. The steps in the pipeline are as follows: (1) medical imaging, (2) image segmentation, (3) multi-sequence MRI selection, (4) feature extraction, (5) model exploration, and (6) model evaluation. Secondly, we evaluated the predictive ability of the radiomics model with accuracy [22,23], stability [19], and efficiency.

In general, we utilized a machine learning-based radiomics method to classify the IDH mutation status of LGG patients, in order to obtain a more accurate, stable, and efficient imaging processing method for classifying tumor subtypes. In this task we followed the “6-Step” general radiomics model to simultaneously tune the combination of four traditional glioma MRI sequences (including T1, T2, FLAIR, and T1Gd) and optimize the settings in the radiomics pipeline. We investigated in detail the predictive ability of different settings on different radiomics pipelines, including multi-sequence MRI selection, image filters, radiomics feature extractors, the data normalization strategy, the dimension reduction approach, the feature selection method, and classifier settings, etc. Based on numerous experiments, we ultimately achieved an optimal pipeline that best distinguished the IDH mutation status.

## 2. Materials and Methods

### 2.1. Data Cohort

**Figure 1:**
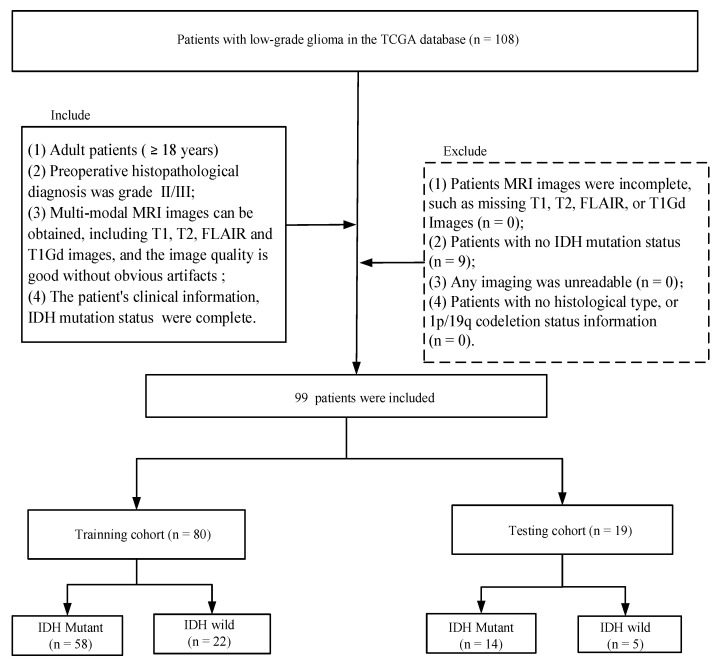
Patient screening flowchart. Abbreviations: TCGA, The Cancer Genome Atlas; T1, T1-weighted; T2, T2-weighted; FLAIR, fluid-attenuated inversion recovery; and T1Gd, post-contrast T1-weighted.

The public retrospective dataset The Cancer Genome Atlas Low-Grade Glioma (TCGA-LGG, https://www.cancer.gov/, accessed on 1 June 2022) was used in this paper, and a total of 108 patients were taken from this public dataset. For each patient, we could easily obtain their molecular status (tumor grade, IDH mutation status, etc.), clinical information (age, sex, etc.), and their MRI sequences. Patients were excluded from the cohort if they met the following criteria: (1) missing T1, T2, FLAIR, or T1Gd sequences (*n* = 0); (2) unavailable for IDH mutation status information (*n* = 9); (3) unavailable for histological type or 1p/19q codeletion status information (*n* = 0); and (4) unreadable for any imaging (*n* = 0). Ultimately, 99 cases (training data, 80 cases; test data, 19 cases) were enrolled in the study (mean age: 46 years; range: 20–76 years) (Figure 1). We can see that most of the patients were IDH-mutant (*n* = 72), while a few patients were IDH-wild (*n* = 27). The histological type has now been reclassified by two pathologists according to 2021 WHO classification [7].

### 2.2. Imaging Data Acquisition

For each of the 108 patients we downloaded 4 3D-MRI sequences from the TCGA-LGG dataset, including T1-weighted (T1), T2-weighted (T2), T1-weighted gadolinium post-contrast (T1Gd), and fluid-attenuated inversion recovery (FLAIR) sequences. These were first gathered from various institutions, and then they were pre-processed under the same anatomical template before being published on The Cancer Imaging Archive (TCIA) platform [24].

In the dataset, we could also get the segmentation mask for each patient. There were two types of segmentation masks available here: the “_GlistrBoost.nii.gz” suffix, referring to the segmentation masks produced by GLISTRboost with the assistance of computers [25], and the “GlistrBoost ManuallyCorrected.nii.gz” suffix. The latter denotes the segmentation masks that were manually corrected after being adjusted using GLISTRboost’s automated segmentation masks [26]. In the current paper, we utilized the manually corrected segmentation masks first, and we used the other type for features extraction.

### 2.3. Radiomics Feature Extraction

Before extracting the radiomic features, BinCount = 25 was used for the discretization, and the MRI sequences were transformed with original, wavelet, and no image filters strategies. Then we extracted radiomic features using the FeAture Explorer (FAE, Version 0.5.2) software for the reproducibility of our results, which is an open-source software package and publicly available tool for radiomics models [27]. The radiomics features we extracted included shape features, texture features and first-order statistical features. A total of 3776 radiomic features were obtained from all 4 of the MRI sequences.

After this, the “label” column was added to the feature matrix file to form a supervised learning matrix and then perform feature preprocessing. The current paper adopts a random classification method, dividing the dataset into a training set and a test set according to a ratio of 8:2 (80 training data and 19 testing data) and removing the invalid features via data cleaning.

### 2.4. “6-Step” General Radiomics Model Exploration

**Figure 2:**
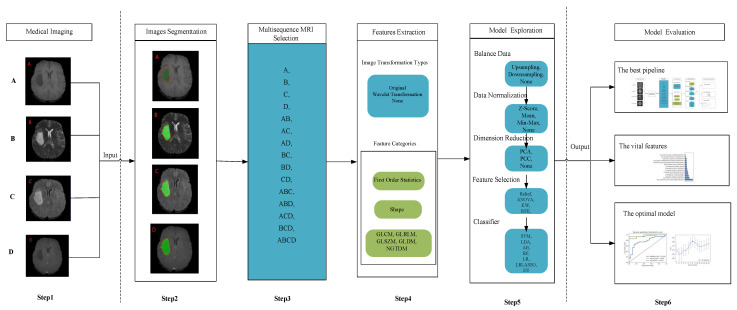
Flow chart of the “6-Step” general radiomics model. The data frames in blue indicate that we chose one of the displayed methods, and the data frames in green mean that we chose all of the displayed methods. A, B, C, and D indicate different medical images. The “+” symbol indicates that different MRI sequences were combined to form a new input object. Abbreviations: GLCM, Gray-level co-occurrence matrix; GLSZM, Gray-level size zone matrix; GLRLM, Gray-level run length matrix; GLDM, Gray-level dependence matrix; and NGTDM, neighboring gray tone difference matrix.

In this section, we propose a “6-Step” general radiomics model, which can investigate both the optimal combined multi-sequence MRI and the most appropriate settings in the radiomics pipeline simultaneously, to best distinguish the IDH mutation status. The “6-Step” model (Figure 2) covers the entire workflow of the radiomics model, including (1), medical imaging, (2) image segmentation, (3) multi-sequence MRI selection, (4) feature extraction, (5) model exploration, and (6) model evaluation. As different medical images are put into the model, different combined multi-sequence MRIs are performed by the “Multi-sequence MRI selection” box. Next, different settings in the radiomics pipeline are investigated in the “feature extraction” and “model exploration” steps. Finally, three important results are shown according to our model evaluation criteria: (1) the best pipeline, (2) the vital features, and (3) the optimal model.

We used four conventional MRI sequences (including T1, T2, FLAIR, and T1Gd) to verify the “6-Step” general radiomics model for predicting the IDH mutation status. We put all 4 sequences into the model, and 15 combined multi-sequence MRIs were generated by the “Multi-sequence MRI Selection” box: T1, T2, FLAIR, T1Gd, T1+T2, T1+FLAIR, T1+T1Gd, T2+FLAIR, T2+T1Gd, T1Gd+FLAIR, T1+T2+FLAIR, T1+T1Gd+FLAIR, T1+T2+T1Gd, T2+FLAIR+T1Gd, and T1+T2+T1Gd+FLAIR. Then we used the radiomics pipeline below for the classification task (Table 1). Firstly, the MRI sequences were transformed with original, wavelet transformation, and none image filters strategies. Secondly, the training set data balance was processed by the random upsampling, downsampling, and none balance methods. Thirdly, the MinMax, Z-Score, mean, and none options were for data normalization. We performed normalization using the training set, then we used the same parameter strategy to normalize the test set. Forthly, principal component analysis (PCA), Pearson correlation coefficients (PCC), and none options were utilized for feature dimension reduction. Fifthly, four feature selection methods were used for comparison, including analysis of variance (ANOVA), Kruskal-Wallis (KW), recursive feature elimination (RFE), and relief. Lastly, seven conventional machine learning classification algorithms were available, including support vector machine (SVM), auto ecoder (AE), random forest (RF), linear discriminant analysis (LDA), logistic regression (LR), logistic regression via lasso (LR-Lasso), and decision tree (DT). A total of 45,360 radiomics pipelines were established, and each optimal model was evaluated by 10 repeated runs. Based on numerous experiments, we reached an optimal pipeline in the “6-Step” general radiomics model for classifying the IDH mutation status.

### 2.5. Model Evaluation

We evaluated the predictive ability of the “6-Step” general radiomics model with regard to the area under the curve (AUC), accuracy, sensitivity, and specificity [22,23]. Meanwhile, stability was evaluated based on the mean and variance of the results of 10 repeated runs [19]. Considering the economic cost and service efficiency, the number of the combined multi-sequence MRIs involved in the model was evaluated for efficiency. In addition, 1–15 features for the radiomics signature were analyzed. All the analyses were evaluated by 5-fold cross-validation on the training data, which was the default setting embedded in the software.

### 2.6. Statistical Analysis

The clinical characteristics of patients and tumor characteristics between the training and testing sets were compared using Student’s *t*-tests. A *p* value of <0.05 indicated statistical significance. Precision-recall (PR) plots and Matthew’s correlation coefficients were used to evaluate the performance of the models. The positive predictive value (PPV) and negative predictive value (NPV) were calculated at the Youden index. The above analyses were performed using R software (R4.2.0) and FAE (Version 0.5.2).

## 3. Results

### 3.1. Clinical Characteristics

A summary of the baseline demographics and clinical features of the research participants is given in Table 2. The train cohort contained 80 patients (58 with mutated IDH and 22 with wild-type IDH), while the test cohort included 19 patients (14 with mutated IDH and 5 with wild-type IDH). There was no significant difference in age (*p* = 0.62), sex (*p* = 0.13), IDH mutation (*p* = 0.92), histological type (*p* = 0.962), or 1p/19q codeletion status (*p* = 0.089), between the train and test cohorts.

### 3.2. Comparison of the Performance of Different Combined Multi-Sequence MRIs Generated by the “6-Step” General Radiomics Model

In Section 2.3 and Section 2.4, we tuned different settings in the “6-Step” general radiomics model, and in this section, we will compare the predictive ability of those different combinations. A total of 45,360 radiomics pipelines were investigated in our model, and the best performance of each combined multi-sequence MRI is shown in Table 3.

**Figure 3:**
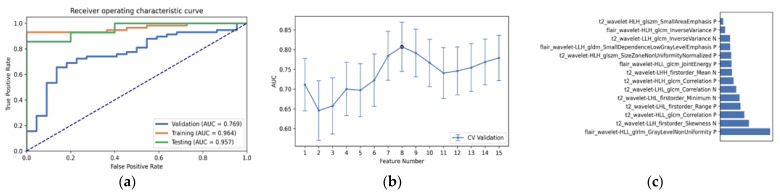
The best performance generated by the “6-Step” general radiomics model was the T2+FLAIR combined multi-sequence with the following settings: wavelet image filter, mean data normalization, PCC dimension reduction, RFE feature selection, and SVM classifier. (**a**) Receiver operating characteristic (ROC) curves of the training, testing, and validation sets; (**b**) the FAE software’s suggestion of a candidate 14-feature model according to the “one-standard error” rule; (**c**) the 14 radiomic features with the highest average feature importance calculated by the best settings with the T2+FLAIR combined multi-sequence.

**Figure 4:**
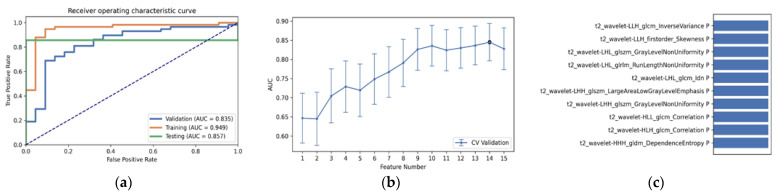
The best diagnostic performance of a single radiomic sequence was T2 with the following settings: wavelet image filter, mean data normalization, PCC dimension reduction, RFE feature selection, and LDA classifier. (**a**) Receiver operating characteristic (ROC) curves of the training, testing, and validation sets; (**b**) FAE software’s suggestion of a candidate 10-feature model according to the “one-standard error” rule; (**c**) the 10 radiomic features with the highest average feature importance calculated by the best settings with the T2 sequence.

**Figure 5:**
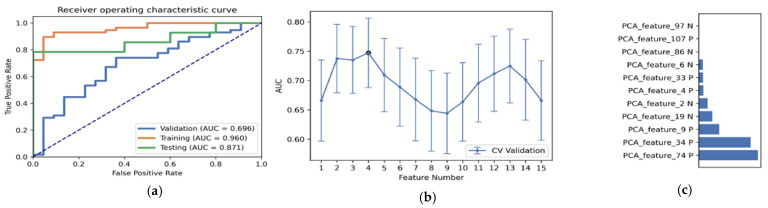
The best diagnostic performance of the three combined multi-sequence was the T1+T2+T1Gd combined multi-sequence with the following settings: wavelet image filter, Z-score data normalization, PCA dimension reduction, RFE feature selection, and RF classifier. (**a**) Receiver operating characteristic (ROC) curves of the training, testing, and validation sets; (**b**) FAE software’s suggestion of a candidate 11-feature model according to the “one-standard error” rule; (**c**) the 11 radiomic features with the highest average feature importance calculated by the best settings with the T1+T2+T1Gd combined multi-sequence.

**Figure 6:**
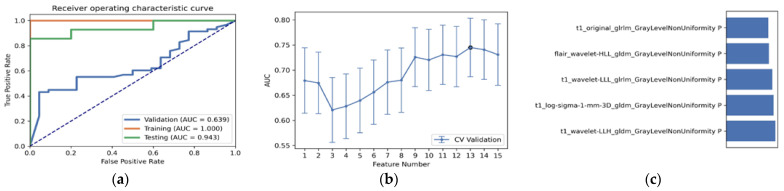
The best performance generated by the T1+T2+FLAIR+T1Gd combined multi-sequence with the following settings: wavelet image filter, mean data normalization, PCC dimension reduction, ANOVA feature selection, and RF classifier. (**a**) Receiver operating characteristic (ROC) curves of the training, testing and validation sets; (**b**) FAE software’s suggestion of a candidate 5-feature model according to the “one-standard error” rule; (**c**) the 5 radiomic features with the highest average feature importance calculated by the best settings with the T1+T2+FLAIR+T1Gd combined multi-sequence.

As shown in Table 3 and Figure 3, the optimal radiomics pipeline for predicting IDH mutation status was the T2+FLAIR combined multi-sequence MRI with specific settings: wavelet image filter, mean data normalization, PCC dimension reduction, RFE feature selection, and SVM classifier. The AUC, accuracy, sensitivity, and specificity were 0.873 ± 0.05 (95% confidence interval [CI], 0.926–1.000), 0.876 ± 0.09, 0.875 ± 0.11 and 0.877 ± 0.15, respectively. The highest AUC was 0.957 (95% CI, 0.875–1.000). The best diagnostic performance of a single radiomic sequence was T2 with the following settings: wavelet image filter, mean data normalization, PCC dimension reduction, RFE feature selection, and LDA classifier, where the AUC, accuracy, sensitivity, and specificity were 0.842 ± 0.05, 0.779 ± 0.08, 0.780 ± 0.13 and 0.780 ± 0.19, respectively (Figure 4). The best diagnostic performance of three combined multi-sequence MRIs was T1+T2+T1Gd with the following settings: wavelet image filter, Z-score data normalization, PCA dimension reduction, RFE feature selection, and RF classifier, with the AUC, accuracy, sensitivity, and specificity being 0.816 ± 0.11, 0.747 ± 0.08, 0.743 ± 0.10 and 0.760 ± 0.20, respectively (Figure 5). The best performance of all four combined multi-sequence MRIs was generated with the following settings: wavelet image filter, mean data normalization, PCC dimension reduction, ANOVA feature selection, and RF classifier. The AUC, accuracy, sensitivity, and specificity were 0.811 ± 0.07, 0.763 ± 0.09, 0.771 ± 0.10 and 0.740 ± 0.16, respectively (Figure 6).

### 3.3. Statistical Result and Feature Analysis

**Figure 7:**
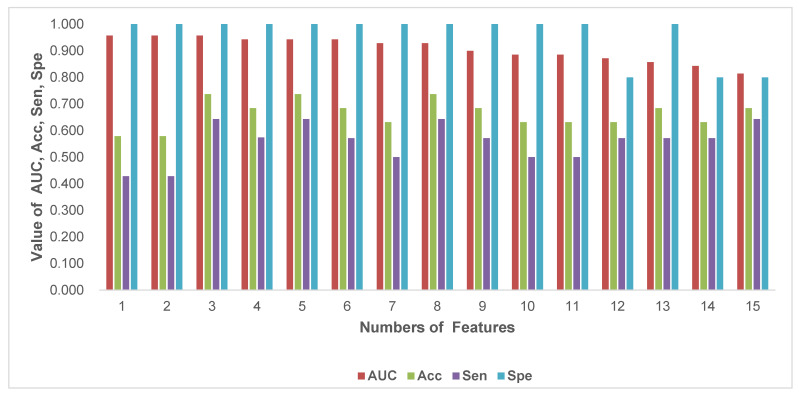
The performance with a different number of features, ranging from 1 to 15. This result was generated by the following radiomic pipeline settings: wavelet image filter, mean data normalization, PCC dimension reduction, RFE feature selection, and the SVM classifier with a number of features ranging from 1 to 15. Abbreviations: AUC, area under the curve; Acc, accuracy; Sen, sensitivity; and Spe, specificity.

The clinical statistics of the best radiomics pipeline is shown in Table 4. To better understand the “6-Step” general radiomics model, we also analyzed the characteristics of the radiomic features retrieved by the optimal radiomics pipeline. For instance, the T2+FLAIR combined multi-sequence had the following settings: wavelet image filter, mean data normalization, PCC dimension reduction, RFE feature selection, and SVM classifier. The radiomic features that had the highest average feature importance are analyzed in Table 5. In addition, we analyzed the performance of the optimal pipeline with a varying number of features ranging from 1 to 15, as shown in Figure 7. In terms of AUC, there was a higher performance of 0.957 when the number of features was 7, 8, and 14. The result with 14 features performed best when AUC, accuracy, specificity, and sensitivity were taken into account.

As shown in Table 5, we gave an explanation to each vital radiomic features, and we found that the top 14 selected features were wavelet transformed features, but not other types. There were 7 gray level co-occurrence matrix (GLCM) features, 4 gray level zone matrix (GLZM) features, and 3 first-order features selected from the most important radiomic features. In light of the statistical results, the gray level co-occurrence matrix (GLCM) feature was the most important radiomic feature.

### 3.4. The Accuracy, Stability and Efficiency of the “6-Step” General Radiomics Model

To eliminate bias induced by a random choice, we averaged the feature significance computed by the experiments using 10 repeated runs for each optimum pipeline of all the combinations, as shown in Table 3. We found the T2+FLAIR combined multi-sequence with wavelet transformation image filter, mean normalization, PCC dimension reduction, RFE feature selection strategy, and SVM classifier was the most accurate, stable, and efficient. This is because the mean and standard deviation of the AUC, accuracy, sensitivity, and specificity of this pipeline were all lower than that of the others. In addition, it only took two MRI sequences for modeling, which can reduce the time needed for the doctor to scan the MRI sequence and make a diagnosis.

## 4. Discussion

In this paper, we investigated the use of “6-Step” general radiomics model—a noninvasive method—in predicting the IDH mutation status. Four traditional glioma MRI sequences (including T1, T2, FLAIR, and T1Gd) and the conventional settings in radiomics pipelines were optimized simultaneously, to obtain a more economical, convenient, accurate, and reliable imaging processing method. Based on 45,360 radiomics pipelines, we arrived at an optimal pipeline for classifying the IDH mutation status, which was the T2+FLAIR combined multi-sequence with wavelet transformation image filter processing, mean normalization, PCC dimension reduction, the RFE feature selection strategy, and the SVM classifier with 14 radiomic features. Our model also determined the most important features calculated by the optimal pipeline for better interpretation of a radiomics model. The grayscale covariance matrix texture (GLCM) features from the T2 and FLARI sequences were of high importance.

In this study, the T1+T2+T1Gd+FLAIR combined multi-sequence was not the best-performing sequence for predicting the IDH mutation status in LGG. This was not surprising, as Kim et al. [14] reported that a multi-parametric MRI radiomics model did not improve the diagnostic performance in IDH mutation status prediction. This might be explained by the fact that a combined multi-sequence can aggregate multi-source information, allowing the radiomics model to learn more information within a given range. Nevertheless, beyond this range, too many features will reduce the performance of the model, as mentioned in [28]. From the perspective of efficiency, this finding will benefit clinical treatment greatly. It can help both in reducing the MRI scanning time for patients and the diagnosis time for doctors. Therefore, using as few MRI sequences as possible to correctly predict the glioma subtypes has great research value.

Previous research has revealed that radiomic features are a reliable means of predicting IDH mutant status [13,14,15,16,17,18,19,20,21,29]. In addition to the conventional MRI sequences, a number of researchers have explored the predictive ability of the T2-FLAIR mismatch for IDH mutation status [30,31,32]. They both confirmed that T2-FLAIR mismatch represented a highly specific imaging biomarker for IDH mutation status. However, the diagnostic performance of different combinations of multi-sequence MRIs and the different settings in the radiomics pipeline were not reported. In our paper, we proposed a “6-Step” general radiomics model, which can simultaneously investigate both the optimal combined multi-sequence MRI and the most appropriate settings in the radiomics pipeline to best distinguish the IDH mutation status. To verify the “6-Step” general radiomics model for predicting the IDH Mutation status, four traditional glioma MRI sequences (including T1, T2, FLAIR, and T1Gd) were used. The predictive ability of the radiomics model was evaluated with regards to accuracy, stability and efficiency. We investigated the predictive ability of different settings in the “6-Step” general radiomics model, including multi-sequence MRI selection, image filters, radiomics features extractors, data normalization strategies, dimension reduction approaches, feature selection methods, and classifier settings, etc. Finally, we achieved an optimal pipeline that best distinguished the IDH mutation status based on numerous experiments. The top 14 radiomic features with the highest average feature importance calculated by the general model were analyzed, revealing that the gray level co-occurrence matrix (GLCM) features with wavelet transformation image filters from the T2+FLAIR multi-sequence combination, are the most important features. Therefore, we recommend that the T2 and FLAIR MRI sequences should be analyzed first during clinical diagnosis of LGG.

Recent studies have demonstrated that segmentation repeatability is essential in terms of feature stability, for it is heavily influenced by different MRI protocols and machines [21,33,34,35,36]. In this paper, we downloaded the segmented data outlined on the TCIA website by using automatic image segmentation and manual supervision, and we performed the experiment using the FAE software, which is a publicly available tool for radiomics models and is applied to many fields [37,38,39,40,41]. Thus, all the experiment results are robust and replicable.

There are several limitations to this study, however. Firstly, because it was retrospective and just a few patients were included, a prospective study with a large cohort of patients is required to validate the stability and repeatability of our findings. We will also use more sufficient data for model validation in the future. Secondly, the diagnostic performance was assessed and verified using information from only a single database, due to the limited number of patient cases in our hospital. Thereforem decisive external validation us required for its clinical application, which should be performed in further studies.

## 5. Conclusions

In this paper, we proposed a “6-Step” general radiomics model to investigate both the optimal combined multi-sequence MRI and the most appropriate settings in radiomics pipelines, which can best distinguish the IDH mutation status. The predictive ability of the general radiomics model was then calculated with regard to accuracy, stability, and efficiency. Several investigations were conducted on the “6-Step” general model, such as multi-sequence MRI selection, image filters, radiomics features extractors, the data normalization strategy, the dimension reduction approach, the feature selection method, and classifier settings, etc. After tuning these settings, a final radiomics pipeline for the prediction of the IDH mutation status was proposed. This paper not only provides a radiomics pipeline which works well for predicting molecular subtypes, but it also contributes to the evaluation of the development of the general model. However, since a small cohort was enrolled in this study, more sufficient data will be used for the proposed model validation in our future study.

## Figures and Tables

**Table 1:** Different options in the main radiomics pipeline of the “6-Step” general model (taking T1, T2, FLAIR, and T1Gd four MRI sequences, for example).

The Main Radiomics Pipeline	Options
Medical images	T1, T2, FLAIR, T1Gd
Combined Multi-sequence MRI	T1, T2, FLAIR, T1Gd, T1+T2, T1+FLAIR, T1+T1Gd, T2+FLAIR, T2+T1Gd, T1Gd+FLAIR, T1+T2+FLAIR, T1+T1Gd+FLAIR, T1+T2+T1Gd, T2+FLAIR+T1Gd, T1+T2+T1Gd+FLAIR.
Images filters	Original/wavelet Transformation/None
Data Balance	Upsampling/Downsampling/None
Normalization	Z- Score/Mean/MinMax/None
Dimension Reduction	PCA/PCC/None
Feature Selection	ANOVA/KW/RFE/Relief
Classifier	SVM/AE/LD/RF/LR/LR-Lasso/DT

45,360 combinations = 15 combined multi-sequence MRI × 3 image filters × 3 data balance methods × 4 data normalization strategies × 3 dimension reduction methods × 4 feature selectors × 7 classifiers. Abbreviations: PCA, principal component analysis; PCC, Pearson correlation coefficients; ANOVA, analysis of variance; KW, Kruskal–Wallis; RFE, recursive feature elimination; SVM, support vector machine; AE, Auto Encoder; LDA, linear discriminant analysis; RF, random forest; LR, logistic regression; LR-Lasso: logistic regression via lasso; and DT, decision tree.

**Table 2:** Clinical characteristics of the train and test cohorts.

Characteristic	Total	Train Cohort	Test Cohort	*p*-Value
Number (%)	99 (100)	80 (80.8)	19 (19.2)	
Age	45.41 ± 13.69	44.88 ± 13.88	47.68 ± 12.98	0.62
Gender Female Male	53 46	39 41	14 5	0.13
IDH status Wildtype Mutation	27 72	22 58	5 14	0.92
Histological type Astrocytomas Oligodendrogliomas NEC	57 14 28	46 11 23	11 3 5	0.962
1p/19q codeletion status No Yes	84 15	68 12	16 3	0.089

*p* < 0.05. Abbreviations: AUC, area under the curve; NPV, negative predictive value; PPV, positive predictive value; and NEC, Not Elsewhere Classified.

**Table 3:** The best performance of each combined multi-sequence MRI generated by the “6-Step” general radiomics model.

Combined Multi-Sequence MRI	Test	Acc	Sen	Spe	Optimal Pipeline
T1	0.778 ± 0.09	0.699 ± 0.15	0.729 ± 0.15	0.720 ± 0.19	Wavelet_MinMax_PCC_RFE_15_RF
T2	0.842 ± 0.05	0.779 ± 0.08	0.780 ± 0.13	0.780 ± 0.19	Wavelet_Mean_PCC_RFE_10_LDA
FLAIR	0.800 ± 0.08	0.742 ± 0.09	0.736 ± 0.11	0.730 ± 0.25	Wavelet_MinMax_Pcc_RFE_15_RF
T1Gd	0.764 ± 0.04	0.679 ± 0.09	0.686 ± 0.16	0.66 ± 0.16	Wavelet_Mean_PCA_RFE_15_LRLasso
T1+T2	0.696 ± 0.12	0.663 ± 0.09	0.714 ± 0.16	0.520 ± 0.22	Wavelet_Mean_PCC_RFE_14_LDA
T1Gd+FLAIR	0.647 ± 0.12	0.658 ± 0.09	0.722 ± 0.11	0.480 ± 0.20	Wavelet_Mean_PCC_RFE_10_LR
T1+FLAIR	0.858 ± 0.08	0.737 ± 0.08	0.686 ± 0.12	0.88 ± 0.16	Wavelet_Mean_PCC_KW_13_AE
T1+T1Gd	0.713 ± 0.09	0.637 ± 0.08	0.636 ± 0.07	0.640 ± 0.17	Wavelet_MinMax_PCC_Relief_15_LDA
T1Gd+T2	0.825 ± 0.09	0.737 ± 0.07	0.786 ± 0.12	0.600 ± 0.24	Wavelet_Zscore_PCC_RFE_14_SVM
T2+FLAIR	0.873 ± 0.05	0.876 ± 0.09	0.875 ± 0.11	0.877 ± 0.15	Wavelet_Mean_PCC_RFE_14_SVM
T1+FLAIR+T1Gd	0.807 ± 0.07	0.763 ± 0.08	0.786 ± 0.12	0.700 ± 0.18	Wavelet_Mean_PCA_RFE_12_AE
T1+T2+FLAIR	0.738 ± 0.14	0.711 ± 0.11	0.714 ± 0.12	0.700 ± 0.18	Wavelet_MinMax_PCC_Relief_3_RF
T2+FLAIR+T1Gd	0.624 ± 0.16	0.663 ± 0.13	0.729 ± 0.17	0.480 ± 0.27	Wavelet_Mean_PCC_RFE_10_LR
T1+T2+T1Gd	0.816 ± 0.11	0.747 ± 0.08	0.743 ± 0.10	0.760 ± 0.20	Wavelet_Zscore_PCA_RFE_10_SVM
T1+T2+T1Gd+FLAIR	0.811 ± 0.07	0.763 ± 0.09	0.771 ± 0.10	0.740 ± 0.16	Wavelet_Mean_PCC_ANOVA_10_RF

Abbreviations: AUC, area under the curve; Acc, accuracy; Sen, sensitivity; and Spe, specificity.

**Table 4:** Clinical statistics in the diagnosis of the best radiomics pipeline (T2+FLAIR combination).

Statistics	Value
Accuracy	0.9357
AUC	0.957
AUC 95% CIs	[0.926–1.000]
NPV	0.8148
PPV	1.0000
Sensitivity	0.9138
Specificity	1.0000

Abbreviations: AUC, area under the curve; NPV, negative predictive value; and PPV, positive predictive value.

**Table 5:** The 14 radiomic features with the highest average feature importance generated by the optimal pipeline (T2+FLAIR multi-sequence combination).

Features	Rank	Description
FLAIR_wavelet-HLL_glrlm_GrayLevelNonUniformity	1	Flair wavelet texture gray region size matrix characteristic gray nonuniformity
T2_wavelet-LLH_firstorder_Skewness	2	T2 wavelet first order characteristic skewness
T2_wavelet-HLL_glcm_Correlation	3	T2 wavelet characteristic correlation of texture gray level co-occurrence matrix
T2_wavelet-LHL_firstorder_Range	4	T2 wavelet texture first-order feature deviation
T2_wavelet-LHL_firstorder_Minimum	5	T2 wavelet texture first-order feature minimum
T2_wavelet-LHL_glcm_Correlation	6	Feature correlation of T2 wavelet texture gray level co-occurrence matrix
T2_wavelet-HLH_glcm_Correlation	7	Feature correlation of T2 wavelet texture gray level co-occurrence matrix
T2_wavelet-LHH_firstorder _Mean	8	T2 wavelet texture first-order feature mean
FLAIR_wavelet-HLL_glcm_JointEnergy	9	Joint energy of flair wavelet texture gray level co-occurrence matrix features
T2_wavelet-HLH_glszm_SizeZoneNonUniformityNormalized	10	T2 wavelet texture gray region size matrix feature normalized region size nonuniformity
FLAIR_wavelet-LLH_gldm_SmallDependence-LowGrayLevelEmphasis	11	Flair wavelet texture gray correlation matrix small dependence low gray emphasis
T2_wavelet-LLH_glcm_ InverseVariance	12	T2 wavelet texture gray level co-occurrence matrix characteristic deficit square
FLAIR_wavelet-HLH_glcm_InverseVariance	13	Characteristic deficit square of gray level co-occurrence matrix of flair wavelet texture
T2_wavelet-HLH_glszm_SmallAreaEmphasis	14	T2 wavelet texture gray area size matrix feature small area emphasis

## Data Availability

The data supporting reported results can be found at the publicly archived datasets TCGA-LGG (https://www.Cancer.gov/ accessed on 30 December 2021).
